# Supplementary material for: DMRforPairs: identifying Differentially Methylated Regions between unique samples using array based methylation profiles
Source: BMC Bioinformatics. 2014 May 15;15:141. doi: 10.1186/1471-2105-15-141 (PMC4046028; doi:10.1186/1471-2105-15-141)
Supplement: Additional file 2 — DMRforPairs output for the comparison of A431-MCF7 and NA17018-NA17105. Please start from the HTML files in each folder. Available via the BMC Bioinformatics website. [file 1471-2105-15-141-S2.zip › 1394847754114233_MOESM2_ESM/A431_MCF7/figures/15180.pdf]

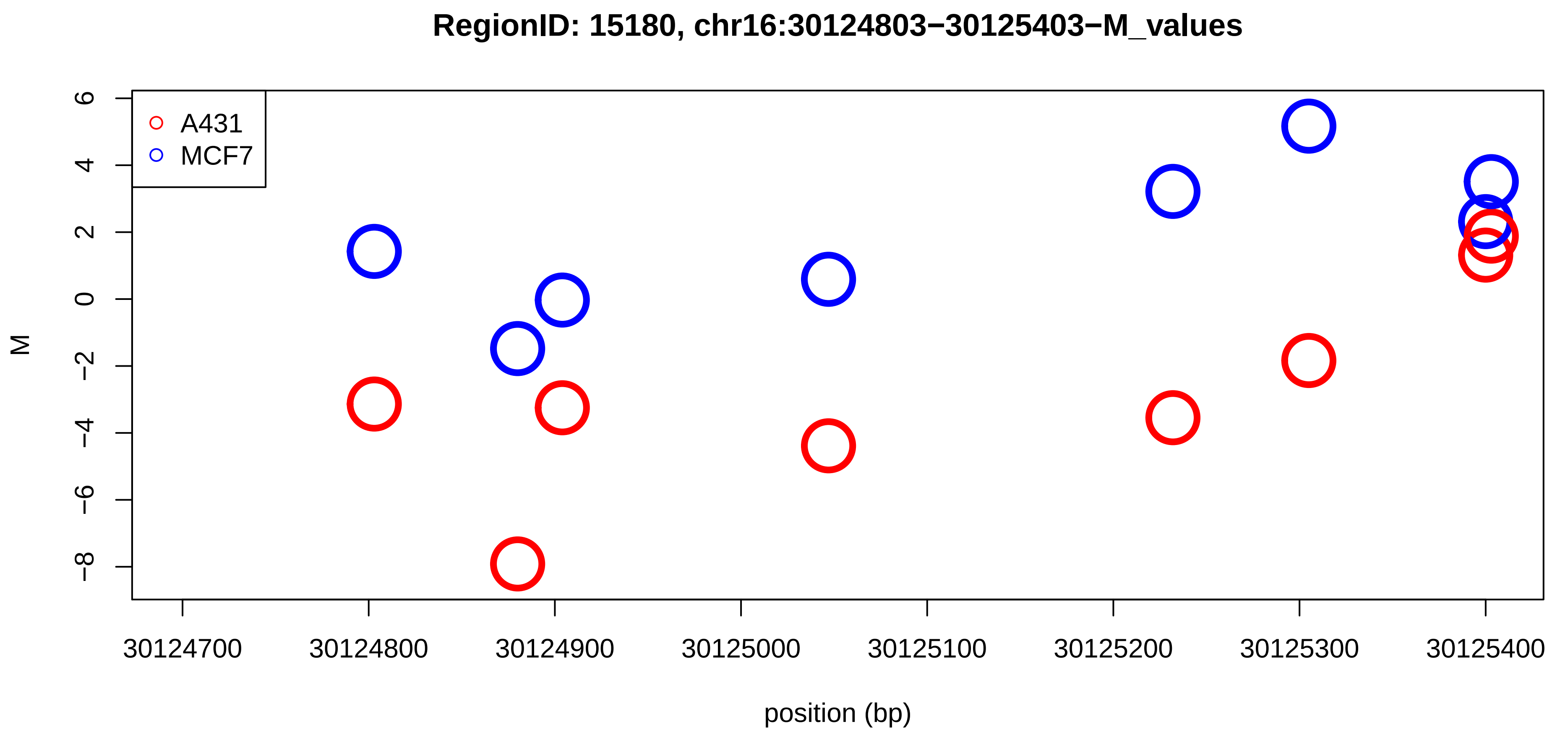
RegionID: 15180, chr16:30124803−30125403−M_values
A431
MCF7
M
position (bp)
30124700
30124800
30124900
30125000
30125100
30125200
30125300
30125400
6
4
2
0
−2
−4
−6
−8

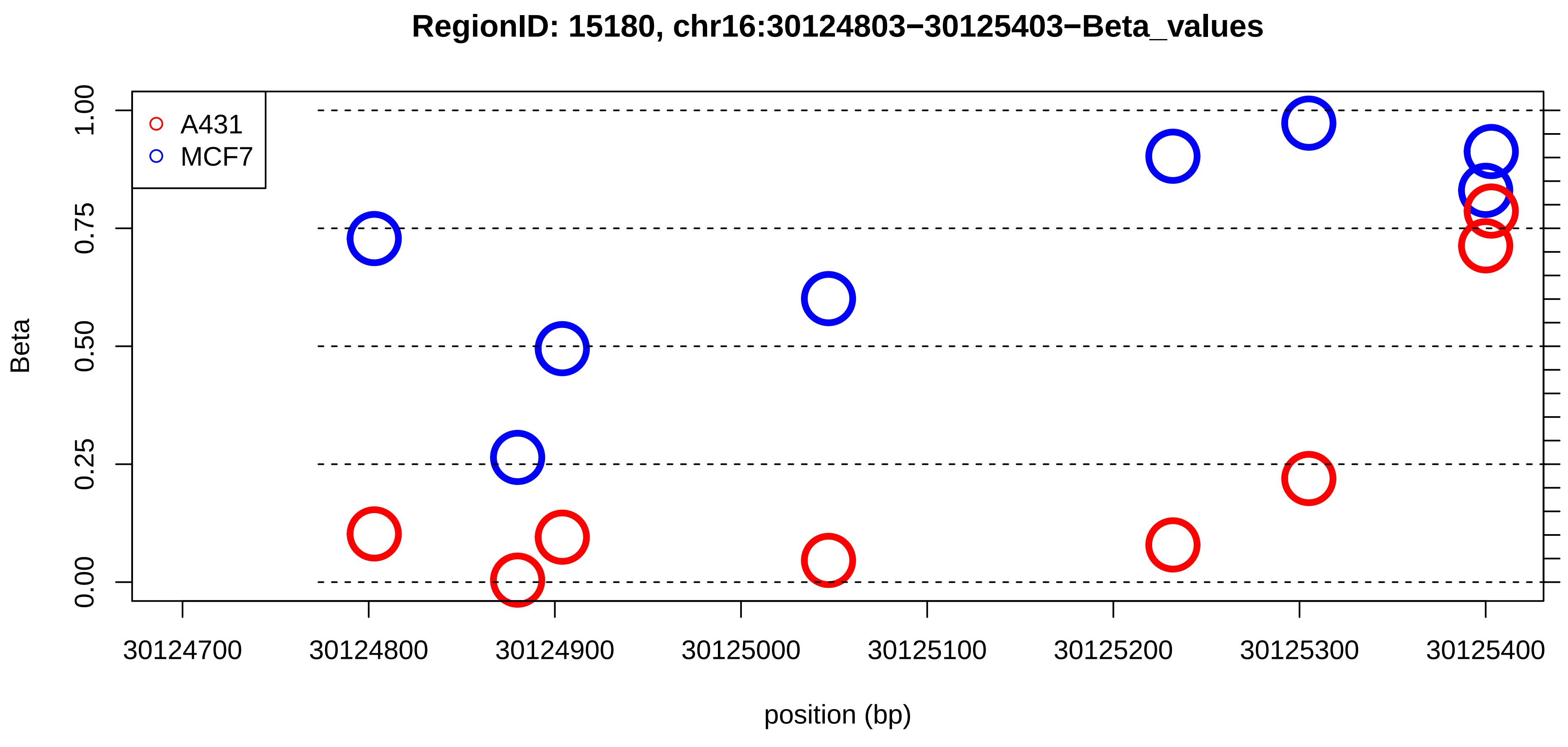
RegionID: 15180, chr16:30124803−30125403−Beta_values
A431
MCF7
Beta
position (bp)
30124700
30124800
30124900
30125000
30125100
30125200
30125300
30125400
1.00
0.75
0.50
0.25
0.00

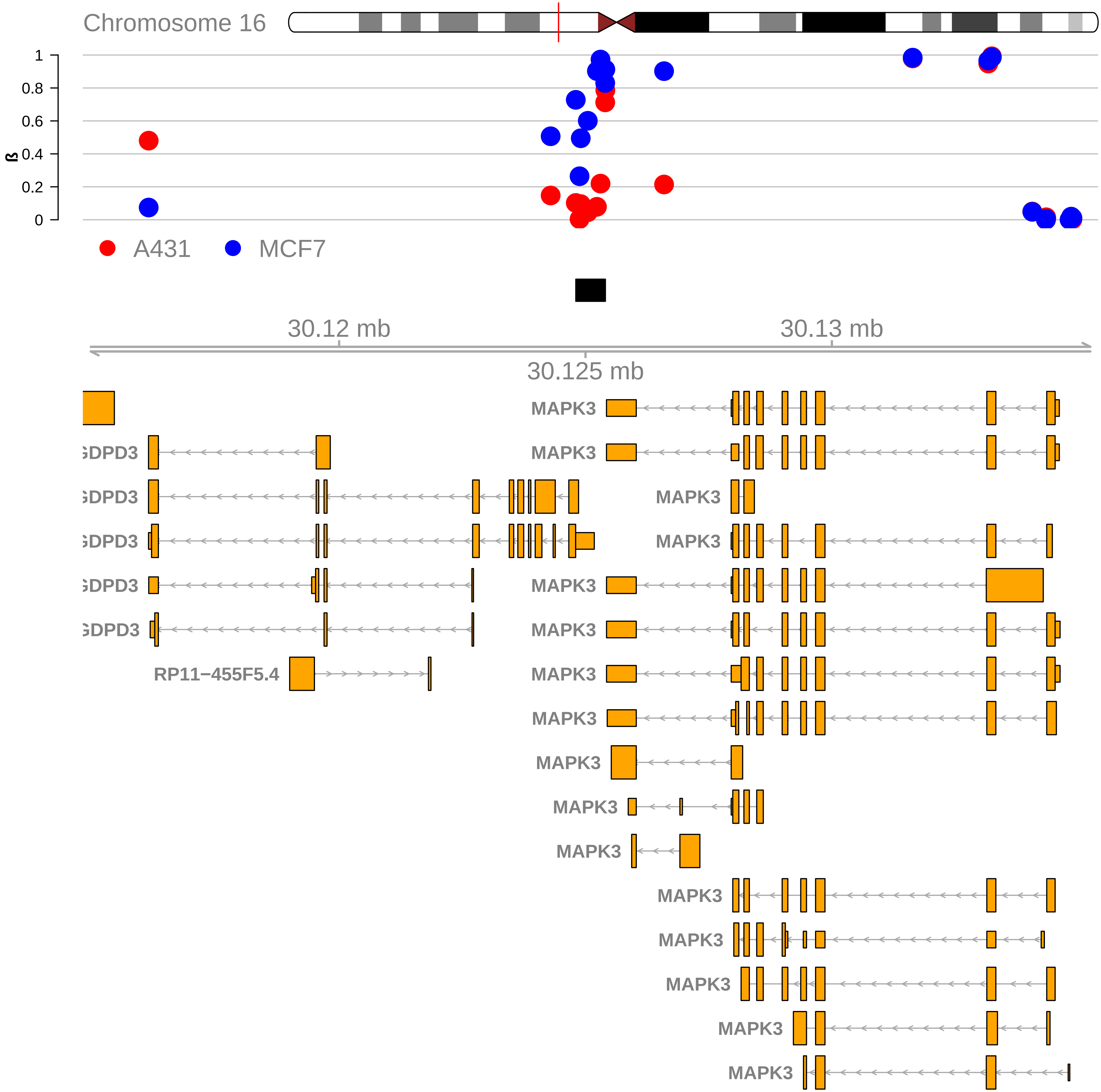

Chromosome 16
1
0.8
0.6
0.4
0.2
0
ß
A431
MCF7
30.12 mb
30.13 mb
30.125 mb
MAPK3
GDPD3
RP11−455F5.4
